# A Multicenter Observational Prospective Cohort Study of Association of the Prehospital National Early Warning Score 2 and Hospital Triage with Early Mortality

**DOI:** 10.1155/2019/5147808

**Published:** 2019-07-01

**Authors:** Francisco Martín-Rodríguez, Raúl López-Izquierdo, Carlos del Pozo Vegas, Juan F. Delgado-Benito, Carmen del Pozo Pérez, Virginia Carbajosa Rodríguez, Agustín Mayo Iscar, José Luis Martín-Conty, Carlos Escudero Cuadrillero, Miguel A. Castro-Villamor

**Affiliations:** ^1^Advanced Clinical Simulation Center, Faculty of Medicine, Universidad de Valladolid, Avda. Ramón y Cajal, 7, 47005 Valladolid, Spain; ^2^Advanced Medical Life Support, Gerencia de Emergencias Sanitarias de Castilla y León, P° Hospital Militar, 24, 47007 Valladolid, Spain; ^3^Emergency Department, Hospital Universitario Rio Hortega, C/ Dulzaina 2, 47012 Valladolid, Spain; ^4^Emergency Department, Hospital Clínico Universitario, Avda. Ramón y Cajal, 3, 47003 Valladolid, Spain; ^5^Department of Statistics and Operative Research & IMUVA, Faculty of Medicine, Universidad de Valladolid, Avda. Ramón y Cajal, 7, 47005 Valladolid, Spain; ^6^Faculty of Occupational Therapy, Speech Therapy and Nursing, Castilla la Mancha University, Avda. Real Fábrica de Seda, s/n, 45600 Talavera de la Reina, Toledo, Spain

## Abstract

**Aim of the Study:**

To evaluate the ability of the prehospital National Early Warning Score 2 scale (NEWS2) to predict early mortality (within 48 hours) after the index event based on the triage priority assigned for any cause in the emergency department.

**Methods:**

This is a multicenter longitudinal observational cohort study on patients attending Advanced Life Support units and transferred to the emergency department of their reference hospital. We collected demographic, physiological, and clinical variables, main diagnosis, and hospital triage level as well as mortality. The main outcome variable was mortality from any cause within two days of the index event.

**Results:**

Between April 1 and November 30, 2018, a total of 1054 patients were included in our study. Early mortality within the first 48 hours after the index event affected 55 patients (5.2%), of which 23 cases (41.8%) had causes of cardiovascular origin. In the stratification by triage levels, the AUC of the NEWS2 obtained for short-term mortality varied between 0.77 (95% CI: 0.65-0.89) for level I and 0.94 (95% CI: 0.79-1) for level III.

**Conclusions:**

The Prehospital Emergency Medical Services should evaluate the implementation of the NEWS2 as a routine evaluation, which, together with the structured hospital triage system, effectively serves to predict early mortality and detect high-risk patients.

## 1. Introduction

Structured triage is a critical process that simplifies decision-making and favors efficient and effective management of a high patient flow [1, 2], making its use in the emergency department (ED) undebatable. However, in the prehospital context, the adaptation of structured triage systems seems to be not as efficient, equally because of the complex and time-consuming application and because of the lack of information in the initial moments, which hinders its applicability outside the hospital setting [3].

The Prehospital Emergency Medical Services (PhEMS) have a growing interest in implementing systems in their operational protocols that allow them to quickly and effectively classify critical patients beyond accidents with multiple victims or catastrophes in which they are already using different specific triage systems designed for this purpose [4, 5].

Today, PhEMS have a wide range of techniques, procedures, and materials for managing multiple pathologies, wherever the patient is. However, their diagnostic capacity is based on an objective and structured clinical evaluation and on a few complementary tests (vital signs, electrocardiogram, and basic analytical determinations) [6]. In this context, recent years have seen the development of different scales that assess the initial severity of patients with prognostic implications called the Early Warning Score (EWS) [4]. Of these, the National Early Warning Score 2 (NEWS2) is the most used internationally and validated in the prehospital context [7, 8]. The NEWS2 has a high predictive capacity [9] and is an excellent tool helping professionals in clinical decision-making [10, 11].

Although the NEWS2 has been evaluated as a predictor of mortality, few prospective studies in the prehospital setting have assessed its usefulness.

The aim of this study was to evaluate the capacity of the prehospital scale NEWS2 in relation to the classification of hospital triage to predict early mortality within 48 hours of the index event. In addition, we explored the performance of this scale in estimating mortality at 7 and 30 days.

## 2. Methods

The study protocol is available online (doi.org/10.1186/ISRCTN17676798). We follow the STROBE statement for reporting.

### 2.1. Ethical Approval

This study was approved by the Research Ethics Committees of all participating centers (reference REC: #PI 18-010, #PI 18-895, and #PI 2018-10/119). All patients (or guardians) signed informed consent.

### 2.2. Source of Data

A prospective multicenter longitudinal observational cohort study was carried out as part of the “*Use of Early Warning Scales in the Prehospital Scope as a Diagnostic and Prognostic Tool*” project of the regional health management of Castilla y León (GRS 1678/A/18), which includes admissions in the Rio Hortega University Hospital and University Clinic of Valladolid, the Hospital Complex of Segovia, and University Assistance Complex of Salamanca, belonging to the public health system of the Autonomous Community of Castilla y León (Spain). The clinical and administrative data included in the database are records of prehospital vital signs, demographic patient data, and level of triage assigned in the ED and mortality (in the hospital).

Prehospital vital signs were recorded in a written document in the place of patient care by the registered nurse (ERN); the remaining data were obtained by reviewing the patients' electronic history 30 days after the index event.

### 2.3. Participants

The study was performed in three provinces of Spain (Valladolid, Salamanca, and Segovia), with a reference population of 886,098 inhabitants, and on all patients attending the five Advanced Life Support (ALS) and referred to its reference hospitals of the public health system between April 1 and November 30, 2018.

Patients were included in the study if they had been evaluated and transferred by an ALS to the ED of the reference hospital and did not meet any exclusion criteria, which were age below 18 years, cardiorespiratory arrest, death on arrival or during transfer, pregnant women, patients with psychiatric pathology or terminal pathology, time of arrival above 45 minutes, and/or patients evacuated by other means of transport or discharged* in situ*.

### 2.4. Outcomes

The main outcome variable was mortality from any cause within two days, stratified by the level of hospital triage assigned in the ED. Mortality was also studied at 7 and 30 days from the index event.

### 2.5. Predictors

At the time of prehospital care, the ERN of each ALS collected the necessary clinical variables for the NEWS2: respiratory rate, oxygen saturation, heart rate, systolic blood pressure, temperature, confusion state (confusion was defined as a score of less than 15 points on the Glasgow Coma Scale), and the use of oxygen [12].

The ERN together with the MD of each ALS, upon arriving at the place of the incident, through the anamnesis directed and the review of the clinical history, determines if the patient presents some type of obstructive pulmonary disease, selecting in this way which respiratory scale of the NEWS2 should be used in each specific case.

The neurological evaluation of each patient was performed conscientiously using the Glasgow Coma Scale, when the patient presented a situation of delirium, was considered not responding consistently to orders, and was categorized with a score in the Glasgow Coma Scale minor of 15 points; therefore it was classified as an alteration of the mental state, classification that coincidences with the AVPU [Alert, Verbal, Pain, Unresponsive] scale.

Temperature was measured using a ThermoScan® PRO 6000 tympanic thermometer (Welch Allyn, Inc., Skaneateles Falls, USA), and measurements of blood pressure, heart rate, and oxygen saturation were made with the LIFEPAK® 15 monitor (Physio-Control, Inc., Redmond, USA) and Corpuls3 (WEINMANN Emergency Medical Technology GmbH, Hamburg, Germany).

In a second time, we collected the demographic variables (sex and age), reason for call, times for arrival, assistance and transfer, prehospital Advanced Life Support maneuvers requiring special follow-up, which include use of intravenous medication or supplementary oxygen, and/or advanced management of the airway (including orotracheal intubation, noninvasive ventilation, and difficult airway). The prehospital primary diagnosis based on the International Classification of Diseases ICD 11 was also collected.

Subsequently, a researcher from each hospital collected the following variables from the electronic history of the patients 30 days after emergency care: mortality from any cause within two days, mortality at 7 and 30 days from the index event, and level of triage assigned in the ED by the Spanish triage system (STS) [13, 14]. This computer program is implemented throughout the public health system of the Autonomous Community of Castilla y León and consists of five levels of prioritization (level I: resuscitation, level II: emergency, level III: urgent, level IV: less urgent, and level V: not urgent).

### 2.6. Missing Data

Prior to applying statistical techniques, the database was cleaned using logical tests, range tests (for detecting extreme values), and data consistency tests. Subsequently, we checked for the presence and distribution of the unknown values of all variables.

### 2.7. Statistical Analysis Methods

All data were stored in an XLSTAT® Biomed database for Microsoft Excel® (version 14.4.0.) and Statistical Product and Service Solutions (SPSS, version 20.0), with which the subsequent statistical analysis was carried out.

Quantitative variables were described as median and interquartile range (IQR) and qualitative variables were described by absolute and relative frequencies.

To compare quantitative variables in two groups, we applied the U-Mann-Whitney test. The Chi-square test was used to study the association between qualitative binary variables. When the frequencies observed in the table discouraged the use of these tests, we used Fisher's exact test.

For the global mortality at 2 days, 7 days, and 30 days, we calculated the area under the curve (AUC) of the receiver operating characteristic (ROC) of the NEWS2 scale and the best score that offered in each case greatest sensitivity and joint specificity. For these scores, we also obtained the positive predictive value (PPV), negative predictive value (NPV), positive likelihood ratio (PLR), and negative likelihood ratio (NLR).

In all the hypothesis tests, we considered significant a p-value of less than 0.05 and chose the usual confidence intervals of 95%.

## 3. Results

### 3.1. Main Findings

**Figure 1:**
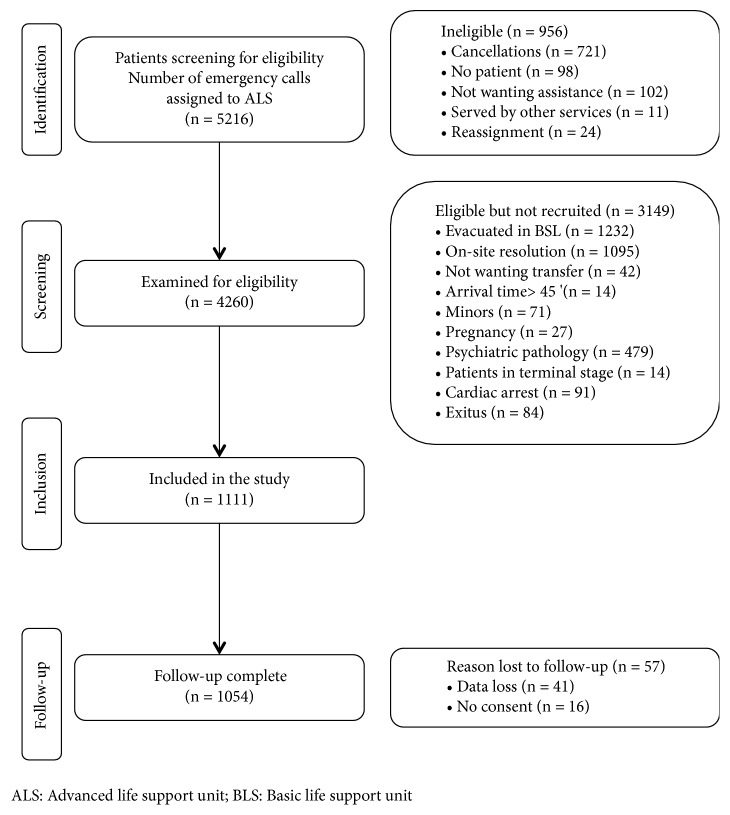
Flow chart of patients recruited to the study.

Between April 1 and November 30, 2018, a total of 1054 patients were included in our study (Figure 1). The median age was 68 years (IQR, 53-81 years), and 418 (39.7%) of the patients were female. The reasons for the demand for care were mostly medical ones with 832 cases (78.9%).

To calculate the NEWS2 during prehospital care, we systematically evaluated all the participants. Nonsurvivors presented a higher respiratory rate, lower oxygen saturation, and an increased presence of confusion with respect to the survivors (p <0.001).

We observed that 90.9% of nonsurvivors needed supplemental oxygen compared to 30.9% in survivors, and 45.5% needed advanced airway management (4.8% in survivors). Regarding the prehospital main diagnosis, processes of cardiovascular origin stood out, 443 cases (42.1%) (Table 1).

### 3.2. Mortality and NEWS2

Early mortality after the index event within the first 48 hours occurred in 55 patients (5.2%), mostly deaths from causes of cardiovascular origin in 23 cases (41.8%). Mortality at 7 days from the index event increased to 81 cases (7.7%) and up to 119 cases (11.3%) at 30 days.

The predictive power of the NEWS2 scale to discriminate mortality at 2, 7, and 30 days is evidenced by an AUC of 0.88 (95% CI: 0.82-0.94), 0.86 (95% CI: 0.81-0.91), and 0.82 (95% CI: 0.77-0.87), verifying how its capacity to assess mortality fell by 6% between the AUC at 2 days and the AUC at 30 days.

### 3.3. Mortality and Spanish Triage System

With respect to early mortality according to the assigned triage priority in the ED, in level I (resuscitation) the mortality rate was 24.4%, in level II (emergency) 5.5%, and in level III (urgency) 0.9%. Table 1 shows the distribution of cases according to the level of triage.

### 3.4. Comparison between NEWS2 and Spanish Triage System

**Figure 2:**
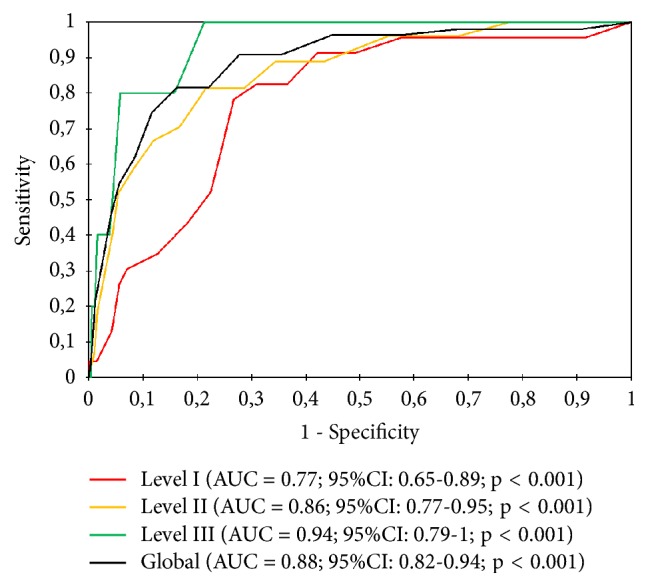
Diagnostic performance curves and areas under the curve with 95% confidence intervals for NEWS and Spanish triage system. Mortality less than 2 days.

When stratified by triage levels, the AUCs of the NEWS2 obtained for short-term mortality varied between 0.77 (95% CI: 0.65-0.89) for level I and 0.94 (95% CI: 0.79 -1) for level III (Figure 2). However, in the analysis of mortality at 7 and 30 days, the best AUC of the NEWS2 has been found for level I with 0.85 (95% CI: 0.76-0.95) for the 7-day mortality and 0.83 (IC 95% CI: 0.74-0.92) for 30 days.

Table 2 shows the best cutoff points in terms of sensitivity and specificity for the NEWS2 according to priority levels and mortality at 2, 7, and 30 days. In the analysis of short-term mortality, we observed that a NEWS2 score greater than or equal to 7 among patients with priority III had a sensitivity of 100 (95% CI 56.6-100) and a specificity of 78.7 (95% CI 75.1-81.9) with a PPV of 4.1 (1.8-9.3) and a NPV of 100 (99.1-100). Meanwhile, in patients with priorities I and II, the cutoff point with better sensitivity and joint specificity rose to 9 points in both cases, with associated NPV of 92.5 (95% CI: 82.1-97.0) for level I and 98.4 (95% IC: 96.2-99.3) for level II (Table 2).

## 4. Discussion

### 4.1. Main Findings

This is the first study evaluating the performance of the joint use of NEWS2 at the prehospital level with the Spanish triage system at hospital level to detect patients at risk of early mortality within 2 days.

Our study shows that the combined use of the NEWS2 and hospital triage can help to identify patients with a high risk of early death, including those that a priori were not emergencies or resuscitation cases.

The capacity decreases progressively in predicting mortality at 7 and 30 days, diminishing its effectiveness globally.

Our results are consistent with those of previous studies [1, 15–20], where the combination of physiological and clinical data from the NEWS2 together with additional data at hospital level during triage in the ED improves the predictive capacity of the studied models.

PhEMS are keen to explore the relationship between diagnostic and prognostic systems, which can help in clinical decision-making* in situ* and in time-dependent diseases. Examples include the Early Warning Score [21, 22], point-of-care testing [23], imaging methods like point-of-care ultrasound [24], or end-tidal carbon dioxide [25]. Among those, the NEWS2 represents a validated and easy-to-use system [7, 26]. Similarly, ED are making an important effort to improve their capacity to adequately classify patients and rapidly detect the most severe cases, for which structured triage systems represent an optimal tool [27–29].

### 4.2. Strengths

This prospective study involved a group of patients already examined by a medical doctor in the place where the emergency occurred at the prehospital level and its subsequent follow-up at hospital level, excluding minor pathologies or those that can be resolved* in situ*. In contrast to other studies, vital signs for the NEWS2 were obtained at the scene or during ambulance transport.

We can find multiple studies on the Early Warning Score in the literature, but we opted for the NEWS2 because it is currently the most used in the PhEMS and has a high bibliographical consistency [8, 30].

### 4.3. Limitations

We used mortality at 2, 7, and 30 days from any cause as the main outcome variable, disregarding deaths outside this window and outside the hospital.

We excluded patients who did not require transport to the hospital or who were evacuated in Basic Life Support units for minor pathologies (after being seen by a medical doctor) to maximize the homogeneity of the patient cohort.

## 5. Conclusion

The combined use of the NEWS 2 and the Spanish triage system presents a very high AUROC, especially for the predictive power of priority III (urgent), increasing the capacity to discriminate those patients with potential to worsen their condition, despite* a priori* being classified otherwise.

In the light of these data, PhEMS should evaluate the routine implementation of the NEWS2 among their procedures, which together with the structured triage system at hospital level effectively serves to predict early mortality and to detect high-risk patients.

## Figures and Tables

**Table 1:** General patient characteristics (death statistics refer to early mortality rates).

	Total	*Survivors*	*Non-survivors*	*p-value*
Number [*n* (%)]	1054 (100)	999 (94.8)	55 (5.2)	
Age (years old) [*Median* (IQR)]	68 (53-81)	67 (53-80)	79 (65-88)	0.001
Gender				
Male [*n* (%)]	636 (60.3)	598 (94.0)	38 (6.0)	
Female [*n* (%)]	418 (39.7)	401 (95.9)	17 (4.1)	0.159
Isochronous (minutes) [*Median* (IQR)]				
Arrival time	10 (8-14)	10 (8-14)	10 (7-13)	0.196
Support time	29 (23-35)	28 (22-35)	33 (25-41)	0.920
Transfer time	10 (7-14)	10 (7-14)	11 (7-18)	0.253
Initial evaluation [*Median* (IQR)]				
Breathing rate (bpm)	18 (14-24)	18 (14-24)	29 (15-34)	0.007
Oxygen saturation (%)	96 (93-98)	96 (93-98)	78 (69-93)	<0.001
Heart rate (bpm)	85 (70-103)	84 (70-102)	93 (72-120)	0.169
SBP (mmHg)	137 (117-157)	137 (119-156)	132 (92 (163)	0.208
Tympanic temperature (°C)	36.4 (36.0-36.9)	36.4 (36.0-36.9)	36.1 (35.4-36.9)	0.355
Mental state (confusion) [*n* (%)]	282 (26.8)	244 (24.4)	38 (69.1)	<0.001
NEWS2 (points) [*Median* (IQR)]	4 (2-8)	4 (2-7)	12 (9-14)	<0.001
Prehospital support [*n* (%)]				
Supplemental oxygen	359 (34.1)	309 (30.9)	50 (90.9)	<0.001
Advanced airway	73 (6.9)	48 (4.8)	25 (45.5)	<0.001
Intravenous medication	915 (86.8)	865 (86.6)	50 (90.9)	0.291
Prehospital diagnostic [*n* (%)]				
Cardiac pathology	443 (42.1)	420 (42.0)	23 (41.8)	
Neurological pathology	192 (18.2)	183 (18.3)	9 (16.4)	
Respiratory pathology	88 (8.3)	82 (8.2)	6 (10.9)	
Injuries and external agents	197 (18.7)	188 (18.8)	9 (16.4)	
Infectious pathology	62 (5.9)	55 (5.5)	7 (12.7)	
Other pathology	72 (6.8)	71 (7.1)	1 (1.8)	<0.001
Hospital triage [*n* (%)]				
Level I: resuscitation	94 (8.9)	71 (7.1)	23 (41.8)	
Level II: emergency	410 (38.9)	383 (38.3)	27 (49.1)	
Level III: urgency	550 (52.2)	545 (54.6)	5 (9.1)	<0.001

IQR: interquartile range; SBP: systolic blood pressure.

**Table 2:** Cut-off points for combined sensitivity and specificity with best score (Youden test) on the NEWS2 scale as a function of mortality (2, 7, and 30 days) and of the assigned triage level.

*n* (%)]	*Mortality*	M2D: 55 (5.2)	M7D: 81 (7.7)	M30D: 119 (11.3)
*Triage*				
Level I 94 (8.9)	*Cut-offs (points)*	9	10	9
	Se % [CI 95%]	82.6 (62.9-93.0)	78.8 (62.2-89.3)	77.5 (62.5-87.7)
	Sp % [CI 95%]	69.0 (57.5-78.6)	82.0 (70.5-89.6)	81.5 (69.2-89.6)
	PPV [CI 95%]	46.3 (32.1-61.3)	70.3 (54.2-82.5)	75.6 (60.7-86.2)
	NPV [CI 95%]	92.5 (82.1-97.0)	87.7 (76.8-93.9)	83.0 (70.8-90.8)
	LR (+)[CI 95%]	2.67 (1.80-3.96)	4.37 (2.49-7.68)	4.19 (2.33-7.50)
	LR (-)[CI 95%]	0.25 (0.10-0.63)	0.26 (0.13-0.51)	0.28 (0.15-0.50)
	OR [IC 95%]	10.58 (3.22-34.77)	16.88 (5.85-48.71)	15.16 (5.51-41.66)
	DA [IC 95%]	72.3 (62.6-80.4)	80.9 (71.7-87.5)	79.8 (70.6-86.7)

Level II 410 (38.8)	*Cut-offs (points)*	9	9	8
	Se % [CI 95%]	81.5 (63.3-91.8)	73.0 (57.0-84.9)	69.6 (56.7-80.1)
	Sp % [CI 95%]	78.3 (73.9-82.2)	79.1 (74.7-82.9)	73.7 (68.9-78.0)
	PPV [CI 95%]	21.0 (14.3-29.7)	25.7 (18.3-34.8)	29.5 (22.4-37.8)
	NPV [CI 95%]	98.4 (96.2-99.3)	96.7 (94.1-98.2)	93.9 (90.4-96.1)
	LR (+)[CI 95%]	3.76 (2.89-4.89)	3.49 (2.64-4.61)	2.65 (2.07-3.39)
	LR (-)[CI 95%]	0.24 (0.11-0.52)	0.34 (0.20-0.59)	0.41 (0.27-0.62)
	OR [IC 95%]	15.90 (5.84-43.27)	10.21 (4.74-21.99)	6.44 (3.47-11.93)
	DA [IC 95%]	78.5 (74.3-82.2)	78.5 (74.3-82.2)	73.2 (68.7-77-2)

Level III 550 (51.1)	*Cut-offs (points)*	7	9	6
	Se % [CI 95%]	100 (56.6-100)	63.6 (35.4-84.8)	69.6 (49.1-84.4)
	Sp % [CI 95%]	78.7 (75.1-81.9)	89.6 (86.7-91.9)	73.1 (69.1-76.7)
	PPV [CI 95%]	4.1 (1.8-9.3)	11.1 (5.5-21.2)	10.1 (6.3-15.8)
	NPV [CI 95%]	100 (99.1-100)	99.2 (97.9-99.7)	98.2 (96.4-99.1)
	LR (+)[CI 95%]	4.70 (4.0-5.52)	6.13 (3.67-10.21)	2.58 (1.90-3.50)
	LR (-)[CI 95%]	0 (0-7.78)	0.41 (0.18-0.89)	0.42 (0.22-0.78)
	OR [IC 95%]	∞ (3.32- ∞)	15.09 (4.28-53.17)	6.20 (2.50-15.38)
	DA [IC 95%]	78.9 (75.3-82.1)	89.1 (86.2-91.4)	72.9 (69.0-76.5)

M2D: mortality 2 days; M7D: mortality 7 days; M30D: mortality 30 days; CI: confidence interval; Se: Sensitivity; Sp: Specificity; PPV: positive predictive value: NPV: negative predictive value; LR: likelihood ratio; OR: odds ratio; DA: diagnostic accuracy.

*∗*Maximum (sensitivity + specificity -1).

## Data Availability

The data will be available upon request.

## References

[1] Ming T., Lai A., Lau P.-M. (2016). Can team triage improve patient flow in the emergency department? a systematic review and meta-analysis. *Advanced Emergency Nursing Journal (AENJ)*.

[2] Shum H. P., Chan K. C., Lau C. W., Leung A. K. H., Chan K. W., Yan W. W. (2010). Triage decisions and outcomes for patients with Triage Priority 3 on the Society of Critical Care Medicine scale. *Critical Care & Resuscitation Journal*.

[3] Tsai L.-H., Huang C.-H., Su Y.-C. (2017). Comparison of prehospital triage and five-level triage system at the emergency department. *Emergency Medicine Journal*.

[4] Ferrandini Price M., Arcos González P., Pardo Ríos M., Nieto Fernández-Pacheco A., Cuartas Álvarez T., Castro Delgado R. (2018). Comparison of the simple triage and rapid treatment system versus the prehospital advanced triage model in multiple-casualty events. *Emergencias*.

[5] Arcos González P., Castro Delgado R., Cuartas Alvarez T. (2016). The development and features of the Spanish prehospital advanced triage method (META) for mass casualty incidents. *Scandinavian Journal of Trauma, Resuscitation and Emergency Medicine*.

[6] Kingswell C., Shaban R. Z., Crilly J. (2017). Concepts, antecedents and consequences of ambulance ramping in the emergency department: A scoping review. *Australasian Emergency Nursing Journal*.

[7] Silcock D. J., Corfield A. R., Gowens P. A., Rooney K. D. (2015). Validation of the National Early Warning Score in the prehospital setting. *Resuscitation*.

[8] Shaw J., Fothergill R. T., Clark S., Moore F. (2017). Can the prehospital National Early Warning Score identify patients most at risk from subsequent deterioration?. *Emergency Medicine Journal*.

[9] Hoikka M., Silfvast T., Ala-Kokko T. I. (2018). Does the prehospital national early warning score predict the short-term mortality of unselected emergency patients?. *Scandinavian Journal of Trauma, Resuscitation and Emergency Medicine*.

[10] Patel R., Nugawela M. D., Edwards H. B. (2018). Can early warning scores identify deteriorating patients in pre-hospital settings? A systematic review. *Resuscitation*.

[11] Williams T. A., Tohira H., Finn J., Perkins G. D., Ho K. M. (2016). The ability of early warning scores (EWS) to detect critical illness in the prehospital setting: A systematic review. *Resuscitation*.

[12] Royal-College-of-Physicians, Royal-College-of-Physicians (2017). *National Early Warning Score (NEWS) 2: Standardising The Assessment of Acute-Illness Severity in the NHS. UpdaTed Report of A Working Party*.

[13] Sánchez Bermejo R., Ramos Miranda N., Sánchez Paniagua A. B. (2016). Ability to predict hospitalization and resource requirements: Comparison of the 3m triage assistance system and the combined spanish triage system and andorran triage model. *Emergencias*.

[14] Gómez-Jiménez J., Gómez-Jiménez J. (2015). *Sistema Estructurado de Triaje - SET: Manual de implementación*.

[15] Ghosh E., Eshelman L., Yang L., Carlson E., Lord B. (2018). Early Deterioration Indicator: Data-driven approach to detecting deterioration in general ward. *Resuscitation*.

[16] Abbott T. E. F., Vaid N., Ip D. (2015). A single-centre observational cohort study of admission National Early Warning Score (NEWS). *Resuscitation*.

[17] Lee Y. S., Choi J. W., Park Y. H. (2018). Evaluation of the efficacy of the National Early Warning Score in predicting in-hospital mortality via the risk stratification. *Journal of Critical Care*.

[18] Abbott T. E. F., Torrance H. D. T., Cron N., Vaid N., Emmanuel J. (2016). A single-centre cohort study of National Early Warning Score (NEWS) and near patient testing in acute medical admissions. *European Journal of Internal Medicine*.

[19] Mapp I. D., Davis L. L., Krowchuk H. (2013). Prevention of unplanned intensive care unit admissions and hospital mortality by early warning systems. *Dimensions of Critical Care Nursing*.

[20] Spagnolli W., Rigoni M., Torri E., Cozzio S., Vettorato E., Nollo G. (2017). Application of the National Early Warning Score (NEWS) as a stratification tool on admission in an Italian acute medical ward: A perspective study. *International Journal of Clinical Practice*.

[21] Kivipuro M., Tirkkonen J., Kontula T. (2018). National early warning score (NEWS) in a Finnish multidisciplinary emergency department and direct vs. late admission to intensive care. *Resuscitation*.

[22] Abbott T. E. F., Cron N., Vaid N., Ip D., Torrance H. D. T., Emmanuel J. (2018). Pre-hospital National Early Warning Score (NEWS) is associated with in-hospital mortality and critical care unit admission: A cohort study. *Annals of Medicine and Surgery*.

[23] Florkowski C., Don-Wauchope A., Gimenez N., Rodriguez-Capote K., Wils J., Zemlin A. (2017). Point-of-care testing (POCT) and evidence-based laboratory medicine (EBLM)–does it leverage any advantage in clinical decision making?. *Critical Reviews in Clinical Laboratory Sciences*.

[24] Press G. M., Miller S. K., Hassan I. A. (2014). Prospective evaluation of prehospital trauma ultrasound during aeromedical transport. *The Journal of Emergency Medicine*.

[25] Hunter C. L., Silvestri S., Ralls G., Stone A., Walker A., Papa L. (2016). A prehospital screening tool utilizing end-tidal carbon dioxide predicts sepsis and severe sepsis. *The American Journal of Emergency Medicine*.

[26] Downey C. L., Tahir W., Randell R., Brown J. M., Jayne D. G. (2017). Strengths and limitations of early warning scores: A systematic review and narrative synthesis. *International Journal of Nursing Studies*.

[27] Kuriyama A., Urushidani S., Nakayama T. (2017). Five-level emergency triage systems: Variation in assessment of validity. *Emergency Medicine Journal*.

[28] Azeredo T. R. M., Guedes H. M., Rebelo de Almeida R. A., Chianca T. C. M., Martins J. C. A. (2015). Efficacy of the manchester triage system: A systematic review. *International Emergency Nursing*.

[29] Chang W., Liu H.-E., Goopy S., Chen L.-C., Chen H.-J., Han C.-Y. (2017). Using the five-level taiwan triage and acuity scale computerized system: factors in decision making by emergency department triage nurses. *Clinical Nursing Research*.

[30] Kievlan D. R., Martin-Gill C., Kahn J. M. (2016). External validation of a prehospital risk score for critical illness. *Critical Care*.

